# MVDT-SI: A Multi-View Double-Triangle Algorithm for Star Identification

**DOI:** 10.3390/s20113027

**Published:** 2020-05-27

**Authors:** Lijian Sun, Yun Zhou

**Affiliations:** Science and Technology on Information Systems Engineering Laboratory, National University of Defense Technology, Changsha 410072, China; sunlijian14@nudt.edu.cn

**Keywords:** multi-view, double-triangle, star identification

## Abstract

Recently, the triangle algorithm has become the most widely used star identification algorithm because of its simplicity and convenience, where the magnitude information plays a key role in the construction of star map features. However, in practice, the magnitude information of the observed star map is often difficult to use, because they might contain errors or be lost in some worst cases. To solve this problem, we proposed a multi-view double-triangle algorithm for star identification in this paper. This algorithm constructs double-triangle features of stars with the angle and distance information of star points. Moreover, to reduce the influence of noise interference on the identification accuracy of the model, we built multi-view double-triangle features for the observed star map to improve the robustness of the algorithm. Synthetic and real experiments show that our algorithm has a high identification accuracy of more than 98.4% in face of “false star” noises and “missing star” noises, and our algorithm is not affected by the focal length and the shooting angle of the star sensor. Moreover, the results also show that our algorithm has good robustness, short identification time and reduced storage costs, which could be beneficial in practice.

## 1. Introduction

Autonomous celestial navigation, also known as astronavigation, is an independent, unconditional and global autonomous navigation system [1], which is widely used in many countries worldwide. The star sensor/tracker is the main part of the celestial navigation system [2], which is an optical measurement device that measures the attitude information of stars using photocells or a camera. The main function of the star sensor includes: star centroid estimation, star identification and attitude calculation. There are lots of star centroid estimation methods [3,4,5], which are applied to calculate the centroid of stars. These methods play an important role in improving the precision of attitude determination for star sensors. Here, the most challenging part is the star identification, which creates the correspondence between observed and cataloged stars [6].

The star identification usually contains two steps: (1) making guide star catalog, (2) designing and implementing identification algorithm, which affects the attitude identification accuracy and attitude output speed of the star sensor. Thus, a good star identification algorithm is very important.

Due to the difference of different application backgrounds, the specific indices of various star sensors are quite different, there is no uniform and recognized star identification evaluation standard up to now. However, as discussed in previous references [6,7,8], a good star identification algorithm usually has high identification accuracy, less identification time, and less storage space (star pattern database, SPD).

The traditional star identification algorithms usually use angular distance and its derivative forms to make the feature of the observed star map, which is relatively simple and generally needs large storage spaces. Moreover, its identification accuracy is generally low, which is not good for practical applications.

In addition, traditional star identification algorithms process most of the information in the observed star map, such as the location of the star centroid and the magnitude information to improve the identification precision. However, when the magnitude information is missing, most of these algorithms cannot identify the observed star map or have poor robustness [9,10]. Therefore, it is necessary to explore a more reliable star identification algorithm which is not based on the magnitude information of the observed star map.

To solve these problems, we proposed a new star identification algorithm named *Multi-View Double-Triangle Algorithm for Star Identification* (MVDT-SI), which can get better robustness, applicability and flexibility. The innovation of this paper is summarized as follows:

1. Our new star identification algorithm does not depend on the magnitude information of the observed star map;
2. The algorithm constructs multi-view double-triangle features to identify stars, which effectively improves the identification accuracy and robustness of the algorithm.
3. Our new star identification algorithm is not affected by the focal length and the shooting angle of the star sensor.

## 2. Related Work

There are two main steps for a star sensor to measure aircraft attitude: centroid estimation and star identification. The first step—centroid estimation—has been well studied. For example, the star centroid location estimation algorithm proposed by Wei [5] reduces the systematic error to less than $3.0\times 10^{-7}$ pixels. Therefore, this paper focuses on the second step—star identification, which is still a challenge.

Star identification algorithms are generally divided into two categories: subgraph-isomorphism-based algorithms and pattern-based algorithms.

The triangle algorithm is one of the most important subgraph-isomorphism-based algorithms. The classical triangle feature identification algorithm was proposed by Junkins [11] in the 1970s to solve the problem of low efficiency and error prone in artificial star identification. Then, Van [12] assigned a probability to each star according to the size of the detected star, so as to select the most reliable triangle. Later, Mortari [13] proposed the pyramid identification algorithm, which introduced a star to construct tetrahedron to solve the “false star” problem. The subgraph-isomorphism-based algorithm also has evolved a voting strategy based on angular distance counting [14]. In 2018, Wang et al. [15] used hash map to accelerate the pyramid identification algorithm. The pyramid identification algorithm has strong robustness to position noises; however, when the false stars increase and the true stars are insufficient, the algorithm may not converge.

Grid algorithm and neural network algorithm are two typical types of pattern-based algorithms. The grid algorithm was first proposed by Padgett in the 1990s [16]. Instead of constructing triangles by various combinations, this algorithm transforms the star map into a grid shape and uses the feature matrix to match. In 2007, Zhang et al. [17] proposed an improved algorithm based on radial and cyclic features, which overcomes the shortcoming of grid algorithms. To overcome the speed problem of step-by-step matching strategy in [17], Zhao et al. proposed K-L transformation to form rough matching and used the star walk procedure to execute to accurately identify stars [18]. Compared with triangle algorithms, the grid algorithm has the advantages of small storage and fast identification speed.

Recently, with the development of artificial intelligence, neural network [19] and other advanced algorithms (such as genetic algorithm [20]) are applied to star identification. The idea of neural network is introduced into the star identification algorithm by Hong [21]. In this algorithm, Hong et al. constructed the feature vector to train the network for star identification. Neural network algorithm has the advantages of high identification accuracy and short identification time. However, the training time of neural network is usually long, which is one of the biggest problems at present. In 2019, a very recent work [22] proposed a star identification network RPNet based on presentation learning to reduce the model training time by simplifying the network structure. Compared with the pattern-based algorithm, the triangle algorithm is widely used and implemented because of its simplicity.

It is worth noting that those above star identification algorithms all select the star centroid location and the magnitude information of the observed star map when constructing the star map features. These algorithms which use the magnitude information of stars [23,24] for selection of the reference star or for identification are more prone to failure because the order of brightness of the stars in the observed star map and the guide star catalog. The studies in [9,10] also suggest that the magnitude information of the stars is an ambiguous feature for comparison and should be avoided. In addition, when the brightness of stars in the observed star map is distorted or missing, we will not be able to accurately obtain the magnitude information of the observed star map. At this time, the above star identification algorithms will be difficult to identify the observed star map accurately.

To solve this problem, other scholars selected reference star based on the distance (between stars and the center of the observed star map) or scan techniques. In 2001, Mortari et al. proposed the pyramid algorithm [25] that does not use star magnitude information. However, it requires additional memory to look for a unique triangle by scanning the triangle combination. To achieve a high speed of identification, some star identification algorithms—low memory SPD [26], optimized SPD (SPOD) [27] were developed specifically. In addition, Kolomenkin et al. proposed geometric voting algorithm (GMV) [28] based on a geometric voting scheme in which a pair of stars in the catalog votes for a pair of stars in the observed star map if the angular distance between the stars of both pairs is similar. In 2018, Deval et al. proposed a star identification algorithm with star ID shortlisting (SISS) [29] which excels at both the identification reliability and speed. However, when there are many false/missing stars in the observed star map, the performance of these algorithms will be significantly reduced.

To address this problem, we proposed an improved algorithm in this paper without considering the magnitude information of the observed star map. The algorithm could have good identification accuracy and robustness by constructing multi-view double-triangle features of the observed star map.

## 3. The Basic Star Catalog

Star catalog is an indispensable part of the star identification based on the star sensor. It can be used to create the triangle feature database of stars for star identification.

The basic star catalog contains tens of thousands of stars, each of which includes the position, brightness, spectrum and other parameters of stars. However, since star sensors can only detect stars in a certain brightness range, we need to process the basic star catalog for star sensors. Star parameters used in star sensors mainly includes the position (right ascension, declination coordinates) and the brightness of stars. We can extract the required parameters according to the limit magnitude in the basic star catalog to create a specific star catalog for the star sensor. This specific catalog is usually called the guide star catalog.

To create the guide star catalog, stars with brightness greater than (magnitude less than) the limit magnitude are selected from the basic star catalog. The star selected in the basic star catalog is called the guide star. Assuming the limit magnitude of star sensor is 6 Mv, 4908 stars with brightness higher than (or equal to) 6 Mv will be selected to form the guide star catalog. After obtaining the guide star catalog, we can match the observed star map with the guide star catalog by comparing the differences between them.

## 4. Multi-View Double-Triangle Algorithm for Star Identification (MVDT-SI)

### 4.1. Coordinate Transformation of the Guide Star Catalog

The celestial sphere is a sphere with infinite radius and the location of stars in the celestial sphere is determined by the right ascension and declination coordinates in the guide star catalog. Therefore, the guide star needs to be projected on the star sensor before identification. When the celestial sphere coordinate system converts to the star sensor coordinate system, their origin can be considered to be coincident. So, the projection of the guide star on the tangent plane of a unit sphere can be considered to be the map on the star sensor.

**Figure 1:**
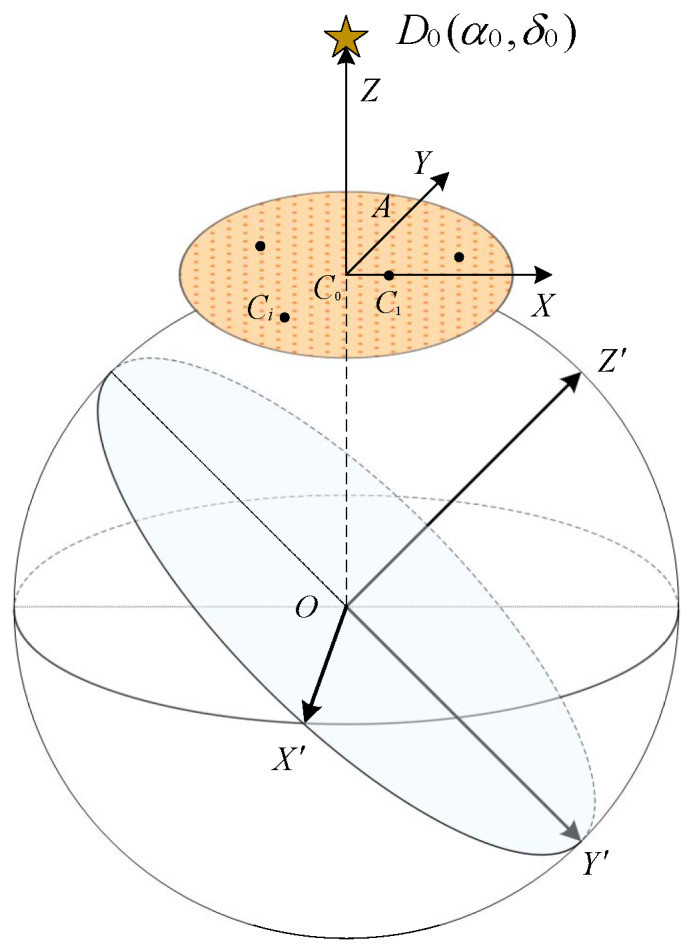
The coordinate transformation from the celestial sphere to the two-dimensional plane.

As shown in the Figure 1, suppose $D_{0}\left(\alpha_{0},\delta_{0}\right)$ is a guide star on the celestial sphere and $C_{0}$ is the projection point of $D_{0}$ on the unit sphere, where $\alpha_{0},\delta_{0}$ is the right ascension and declination coordinates of star $D_{0}$ respectively. In the unit sphere coordinate system, the $C_{0}$ point $\left(\alpha_{0},\delta_{0}\right)$ can be expressed as:

(1) $$\left(\begin{array}{c} x_{0}\\ y_{0}\\ z_{0} \end{array}\right)=\left(\begin{array}{c} \cos\alpha_{0}\cos\delta_{0}\\ \sin\alpha_{0}\cos\delta_{0}\\ \sin\delta_{0} \end{array}\right)$$

In this case, the tangent plane of unit sphere at $C_{0}$ point can be expressed as:

(2) $$\cos\alpha_{0}\cos\delta_{0}x+\sin\alpha_{0}\cos\delta_{0}y+\sin\delta_{0}z=1$$

where $\left(x,y,z\right)$ is any point on the tangent plane. For any star $D_{i}\left(\alpha_{i},\delta_{i}\right)$ except $D_{0}$ in the guide star catalog, a ray can be drawn from the center of the sphere *O* to $D_{i}$. The parameter equation is as follows:

(3) $$\left\{\begin{array}{c} x=t\cos\alpha_{i}\cos\delta_{i}\\ y=t\sin\alpha_{i}\cos\delta_{i}\\ z=t\sin\delta_{i} \end{array}\right.$$

When the ray is not parallel to the tangent plane, the intersection point $C_{i}$ of the ray and the tangent plane can be calculated by the joint Equations (2) and (3). At this time, the intersection point $C_{i}$ is the projection point of the guide star $D_{i}$ on the tangent plane with $C_{0}$ as the center.

When all the stars are projected to the tangent plane, the number of stars in the tangent plane can be adjusted according to the field of view (FOV) of star sensor. Let *S* be the FOV of star sensor and

(4) L=2πS/360

On the tangent plane, a circle with a radius of $L/\sqrt{2}$ can be made with $C_{0}$ as the center. At this time, all the projection star points in the circle make the set *C*. Obviously, the stars contained in *C* are all guide stars in the star sensor of which center is reference star $D_{0}$ and the FOV is *S*.

To make the reference star point $C_{0}$ and the set *C* in a unique coordinate system, we defined the plane coordinate system by guide stars in the guide star catalog. In other words, we selected the star $C_{1}$ (which is the closest point to the $C_{0}$ in *C*) as the auxiliary star to establish the coordinate system. In this case, we can establish a new coordinate system, where the vector $\vec{C_{0}C_{1}}$ is the *X* axis, the $\vec{C_{0}C_{1}}$’s counter-clockwise orthogonal vector $\vec{C_{0}A}$ on the tangent plane is the *Y* axis, and the vector $\vec{C_{0}D_{0}}$ is the *Z* axis. Where

(5) $$\vec{C_{0}A}=\vec{C_{0}D_{0}}\times \vec{C_{0}C_{1}}$$

The star points in the set *C* can be represented in the new coordinate system XYZ. In this coordinate system, the coordinates of all star points on the *Z* axis are always 0. In other words, all the stars in the set *C* are projected onto the $C_{1}C_{0}A$ two-dimensional plane. Finally, the coordinates of all stars in the plane are standardized with the module of $\vec{C_{0}C_{1}}$ as the unit length.

For each reference star *D* in the guide star catalog, we can calculate the coordinates of star points in the FOV *S*. Therefore, we can get the projection map of all guide stars on the star sensor based on different reference stars.

### 4.2. Rotation Invariance of the Observed Star Map

After the star map captured by the star sensor is denoised (centroid estimation, etc.), the star points on the star map often have different coordinates because of the different shooting angles of the star sensor. To ensure that our algorithm is not affected by the shooting angle of the star sensor (rotation invariance), we defined the plane coordinate system by selecting stars from the observed star map.

**Figure 2:**
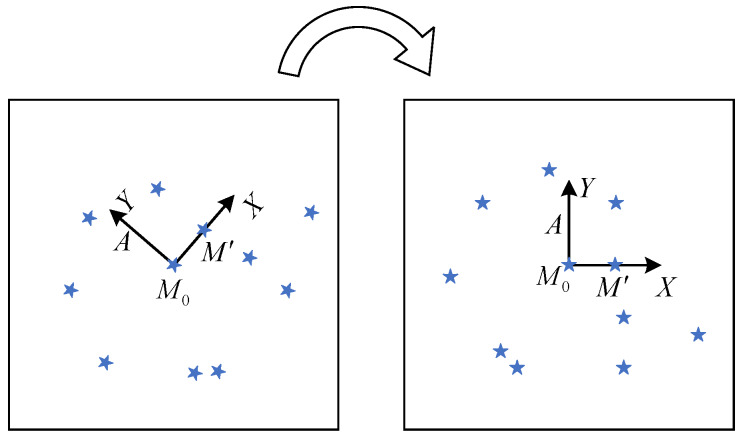
The establishment of the coordinate system of the observed star map.

First, we selected a star as the reference star from the observed star map, then we found the auxiliary star near the reference star. Next, we can construct the *X*-axis by the vector composed of the reference star and the auxiliary star and the *Y*-axis by its counter-clockwise orthogonal vector. After defining the coordinate axis and unit length, the star map has rotation invariance. At the same time, when the reference star and auxiliary star are determined, the observed star map is unique. As shown in the Figure 2, this process could be summarized as follows:

1. We selected a star in the observed star map randomly named the reference star $M_{0}$, and then we selected the auxiliary star $M^{\prime }$ according to certain rules (as shown in Section 4.3);
2. We took the direction of $\vec{M_{0}M^{\prime }}$ vector as *X*-axis and its counter-clockwise orthogonal vector $\vec{M_{0}A}$ as *Y*-axis to establish a new coordinate system;
3. The coordinates of all stars in the observed star map are represented in the new coordinate system;
4. We standardized the coordinates of all stars in the new coordinate system by taking the module of vector $\vec{M_{0}M^{\prime }}$ as unit length.

With the above method, we can obtain the unique coordinates of the observed star map with the reference star $M_{0}$ as the center and $M^{\prime }$ as the auxiliary star. When we select different reference stars and auxiliary stars, we can get the coordinate representations of the observed star map under different views, thus we can generate the observed star map features from multiple views.

### 4.3. Multi-View Double-Triangle Features of the Observed Star Map

In practical engineering applications, the magnitude information of the observed star map captured by star sensor is often missing or noisy. Therefore, our MVDT-SI algorithm excludes the magnitude information and uses the coordinate information of the centroid of the stars in the observed star map when constructing features.

After preprocessing of guide stars in the guide star catalog and observed stars in the observed star map (as discussed in Section 4.1 and Section 4.2), the determination of reference stars and auxiliary stars means that the unit length and coordinates of stars in each observed views are unique. Therefore, in one observed views, the three angle and edges of each triangle can be constructed to generate a fixed vector, so we can use the triangle to construct the features of the observed view.

**Figure 3:**
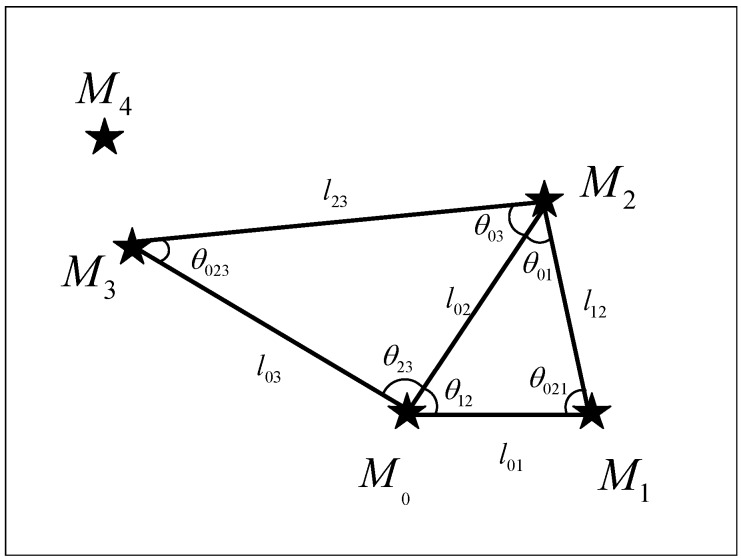
Illustration of the double-triangle feature of the observed star map in the first view. Four stars located at the two-dimensional coordinate system form the double-triangle feature. Each vertex of the triangle is a star, and each edge length of the triangle is the distance between two stars in the two-dimensional plane.

First, as shown in the Figure 3, we randomly selected a star as the reference star $M_{0}$ in the observed star map and calculated the distance between the reference star $M_{0}$ and other stars in the observed star map. Next, we selected the nearest four points, which are $M_{1},M_{2},M_{3},$ and $M_{4}$ in sequence and used those five points to construct double-triangle features of multiple views.

When different auxiliary stars are selected, multiple groups of triangles can be constructed to describe the features of the observed star map. So we constructed the triangle features in multiple views to reduce the impact of noise points on the observed star map.

After the reference star and the auxiliary star are selected, the unit length and coordinates of stars in the observed view are unique. In other words, the triangles in the observed star map and the triangles in the corresponding guide star catalog are congruent. Thus, we can construct the double-triangle features of multiple views by using the “angle-angle-angle” and “edge-edge-edge” information in different triangles.

For the first view, we selected $M_{1}$ as the auxiliary star and used the star sets $\left\{M_{0},M_{1},M_{2}\right\}$ and $\left\{M_{0},M_{2},M_{3}\right\}$ to construct double triangles. Let θ represent the angle of the triangle and *l* represent the edge length of the triangle. At this point, we can construct the double-triangle feature in the first view as $F_{1}=\left[\theta_{01},\theta_{021},\theta_{12},\theta_{023},\theta_{03},\theta_{23},l_{01},l_{02},l_{12},l_{02},l_{03},l_{23}\right]$.

When there is a noise point near the reference point, the feature of the first view constructed by the auxiliary point $M_{1}$ is easily destroyed by noise. Therefore, the feature of the second view should be constructed. So we selected $M_{2}$ as the auxiliary point and used the star sets $\left\{M_{0},M_{2},M_{3}\right\}$ and $\left\{M_{0},M_{3},M_{4}\right\}$ to construct the double-triangle feature, i.e., $F_{2}=\left[\theta_{02},\theta_{032},\theta_{23},\theta_{034},\theta_{04},\theta_{34},l_{02},l_{03},l_{23},l_{03},l_{04},l_{34}\right]$.

To reduce the interference of noise points on the observed star map, we select different reference stars to generate more double-triangle features of different views. By using the multi-view double-triangle features of the observed star map, our algorithm can achieve good robustness and high identification accuracy.

### 4.4. Features of the Guide Star Catalog

**Figure 4:**
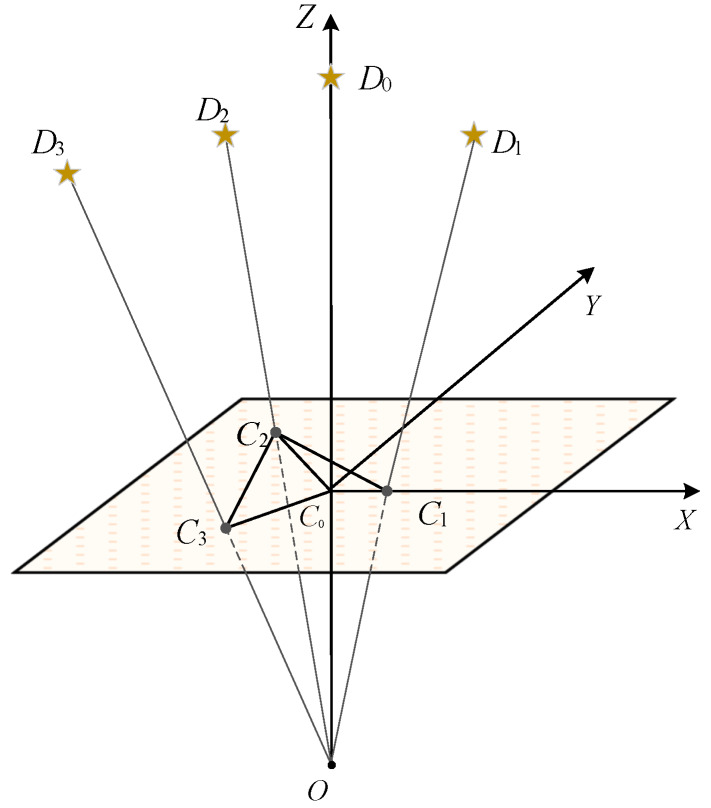
The projection map of the guide star on the star sensor with $D_{0}$ as reference star.

As shown in the Figure 4, through the preprocess in Section 4.1, we can represent stars in the guide star catalog in the two-dimensional plane coordinate system. Thus, we can use the star points in the two-dimensional plane to construct the double-triangle features for reference stars in the guide star catalog. Since there are no noise points in the guide star catalog, we constructed the star features in the first view. By calculating and sorting the distance from each star point in the two-dimensional plane to the reference star $D_{0}$, we can find the three closest points $C_{1},C_{2},C_{3}$ from the reference star $C_{0}$ ($C_{0}$ is the projection point of $D_{0}$ on the star sensor). Thus, the guide star feature of the reference star $D_{0}$ is $H=[\theta_{01},\theta_{021},\theta_{12},\theta_{023},\theta_{03},\theta_{23},l_{01},l_{02},l_{12},l_{02},l_{03},l_{23}]$. Therefore, when there are *K* stars in the guide star catalog, we can get the double-triangle feature database $\left\{H_{1},H_{2},\cdots ,H_{K}\right\}$.

### 4.5. MVDT-SI Algorithm Flow

**Figure 5:**
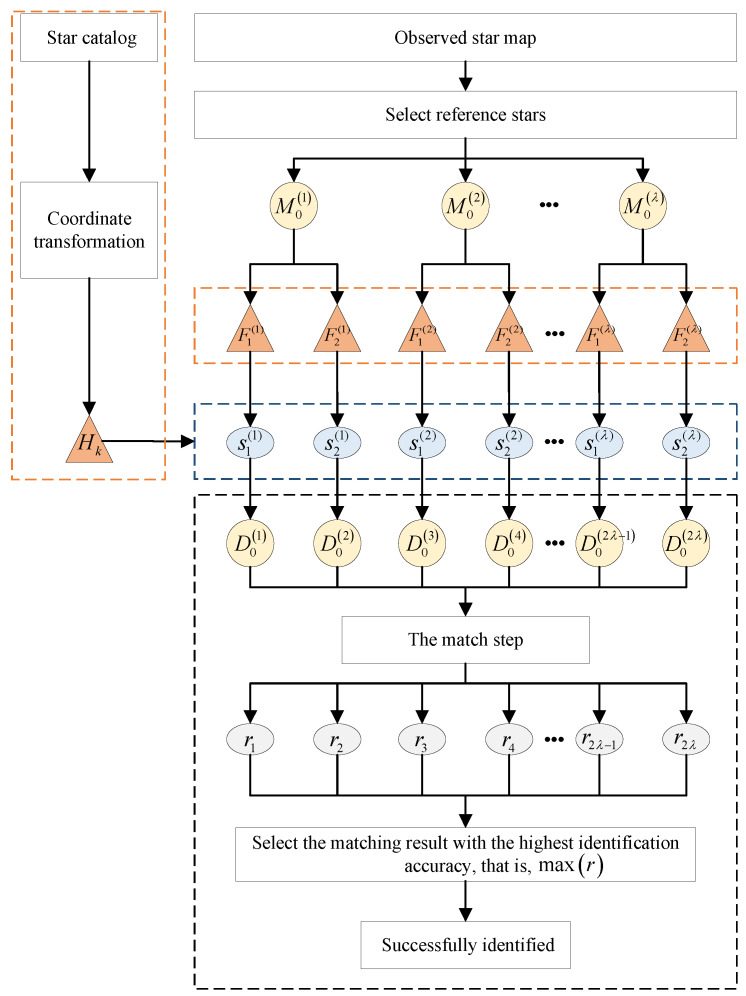
Multi-View Double-Triangle Algorithm for Star Identification Flow.

**Figure 6:**
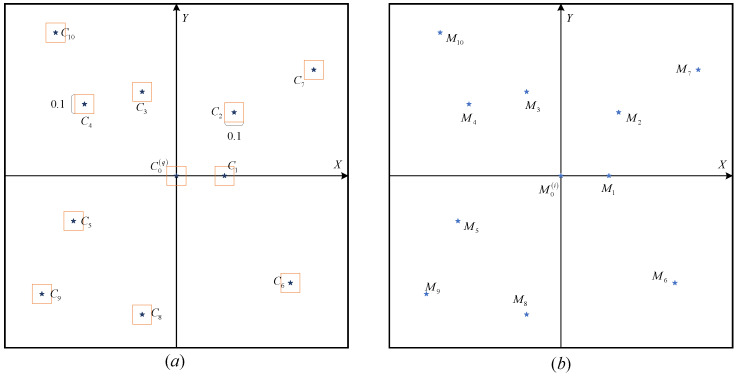
Star point number matching in the observed star map. (**a**) The standardized star map of the guide star map. (**b**) The standardized star map of the observed star map.

As shown in the Figure 5, the MVDT-SI algorithm flow is summarized as follows:

1. Suppose there are *N* star points on the observed star map, and λ is the maximum number of the selected reference stars. If N<4, the observed stars cannot be identified; If 4≤N≤λ, one star point by one on the observed star map is used as the reference star to construct the double-triangle feature; If N>λ, we randomly selected λ star points as the reference stars and constructed the double-triangle features, i.e., the set of reference stars is $\left\{M_{0}^{\left(1\right)},M_{0}^{\left(2\right)},\cdots ,M_{0}^{\left(\lambda \right)}\right\}$.
2. The second step is to identify the reference stars in the observed map. For the *i*-th reference star $M_{0}^{\left(i\right)}\left(i=1,2,\cdots ,\lambda \right)$, their double-triangle features $F_{j}^{\left(i\right)}\left(j=1,2\right)$ are constructed based on different auxiliary stars $M_{1}^{\left(i\right)}$ and $M_{2}^{\left(i\right)}$ respectively. In other words, each reference star $M_{0}^{\left(i\right)}$ has features of double views. Then, we compared the feature information $F_{j}^{\left(i\right)}$ on different views of the observed star map with the feature $H_{k}\left(k=1,2,\cdots ,K\right)$ in the feature database $\left\{H_{1},H_{2},\cdots ,H_{K}\right\}$. In other words, we calculated the sum of the differences between their features, i.e.,
  (6) $$s_{j}^{\left(i\right)}=\min_{k=1,2,\cdots ,K}\left(\sum_{c=1}^{6}\left(\left|\Delta \theta_{c}\right|+\Delta l_{c}\right)\right),j=1,2$$
  To reduce the computational complexity of $s_{j}^{\left(i\right)}$, we only select $K_{S}$ stars in the feature database $\left\{H_{1},H_{2},\cdots ,H_{K}\right\}$ for matching. $K_{S}$ stars that meet the following conditions will be selected:
  (7) $$l_{H_{k_{s}}}-l_{F_{r}}<\min\left(\min_{k=1,2,\ldots ,K}\left(l_{H_{k}}-l_{F_{r}}\right)*200,50\right),\;k_{s}=1,2,\ldots ,K_{s}$$
  (8) $$l=\sum_{c=1}^{6}l_{c}$$
  where $l_{F_{r}}$ is the sum of the edge length feature of the reference star in the observed star map, $l_{H_{k}}$ is the sum of the edge length feature of the *k*-th star in the feature database, and $l_{H_{k_{s}}}$ is the sum of the edge length feature of the $k_{s}$-th selected star in the feature database. $\min_{k=1,2,\ldots ,K}\left(l_{H_{k}}-l_{F_{r}}\right)$ represents the minimum value of the difference between the sum of the edge length of the reference star in the observed star map and the star in the feature database $\left\{H_{1},H_{2},\cdots ,H_{K}\right\}$. After selection, $K_{S}$ is only about 3% of *K* (the number of guide star catalog). At this time, the selected feature database is $\left\{H_{1},H_{2},\cdots ,H_{K_{s}}\right\}$ and the $s_{j}^{\left(i\right)}$ is calculated as follows:
  (9) $$s_{j}^{\left(i\right)}=\min_{k=1,2,\cdots ,K_{s}}\left(\sum_{c=1}^{6}\left(\left|\Delta \theta_{c}\right|+\Delta l_{c}\right)\right),j=1,2$$
  Next, we respectively selected the corresponding star number with the smallest difference $s_{j}^{\left(i\right)}$ in the guide star catalog as the number of $M_{0}^{\left(i\right)}$ star in the observed star map. At this time, we got the most likely stars $\left\{D_{0}^{\left(1\right)},D_{0}^{\left(2\right)},\cdots ,D_{0}^{\left(2\lambda \right)}\right\}$ corresponding to different reference stars $\left\{M_{0}^{\left(1\right)},M_{0}^{\left(2\right)},\cdots ,M_{0}^{\left(\lambda \right)}\right\}$ on the $2\lambda \left(2N\right)$ views.
3. The third step is to identify all the star points in the observed map based on the reference stars. Based on the second step, we can obtain the number of the reference star $M_{0}^{\left(i\right)}$ in the observed star map which is corresponding to the star $D_{0}^{\left(q\right)}$ (the projection point is $C_{0}^{\left(q\right)}$, q=1,2,⋯,2λ) in the guide star catalog. Therefore, through the operation of the Section 4.1 and Section 4.2, we can get the standardized star maps of the guide star map and the observed star map. First, as shown in the Figure 6, we created boxes (size 0.1×0.1 unit) whose centers are stars in the guide star map. Next, we determined whether the stars in the observed star map are in these boxes. If one star point on the observed star map falls into a box of the guide star map, the star point will be marked with the corresponding number of the guide star. If the star point falls into multiple boxes at the same time, the point is marked with the corresponding number of the guide star with the smallest magnitude within the box. Otherwise, it is marked as −1.
4. Finally, the star identification results *r* of different star points in different views are calculated. The statistical value of star identification result *r* is increased by 1 for each successful star identification. In other words, the star identification results *r* is the number of star points on the observed map which is not marked as −1. According to the identification results of reference stars in different views, the results with the most successful star identification is selected as the final number of the star points in the observed star map.

## 5. Experiments

### 5.1. Experiment Settings

In this paper, we proposed a star identification algorithm based on multi-view double triangles and named it MVDT-SI. State-of-the-art star identification algorithms which claim to provide high robustness and fast identification were implemented. We compared a high identification accuracy algorithm with star ID shortlisting (SISS) [29], a high speed algorithm based on search tree with optimized database (STOD) [27], and a highly robust geometric voting algorithm (GMV) [28] with our proposed algorithm MVDT-SI. In addition, we compared our MVDT-SI algorithm with following algorithm settings:

Single-view single-triangle algorithm for star identification (SVST-SI): This algorithm uses a single triangle in the first view of the reference star and the auxiliary star to construct the feature of the observed star map. In other words, the SVST-SI algorithm uses a triangle consisting of $\left\{M_{0},M_{1},M_{2}\right\}$ to construct the feature of the reference star with λ=1.

The star identification algorithm is used to capture the attitude of star sensors in the real world, thus its identification time should be short and the identification accuracy should be high. Moreover, because the storage capacity of the star sensor is limited, the star identification algorithm must consider the space complexity.

Additionally, there always are many interferences in the real observed star map. Therefore, the robustness should be measured by the star identification algorithm under certain interference conditions. Generally speaking, the interference can be divided into interference noises and interference stars.

Interference noises refer to the position noises and magnitude noises of stars. The position deviations of a star point mainly comes from the calibration errors of the star sensor (such as focal length measurement errors, lens distortions, optical axis deviation errors, etc.) and the errors of the star point position algorithm. The magnitude noises show the sensitivities of the star sensor to the brightness of stars.

There are two kinds of interference stars, one is “false star”, such as planets, nebular dust, space debris, etc. It is difficult to distinguish its imaging target from ordinary star point target from the observed star map. In addition, due to the limited resolution of the star sensor to the magnitude, some stars with weak brightness might also be captured, but they cannot find the corresponding match from the guide star catalog. Another kind of interference star is “missing star”, i.e., the star that should have been captured does not appear in the observed map for some reason.

**Figure 7:**
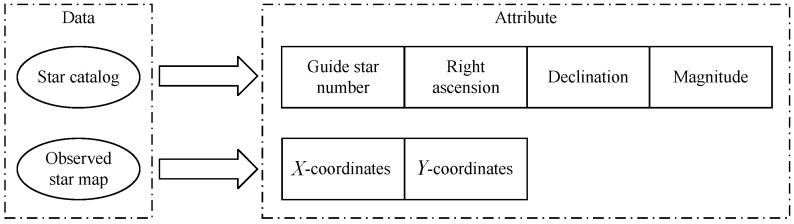
Data attributes of the guide star catalog and the observed star map.

In this experiment, all algorithms are tested on a desktop processor with a clock speed of 2.5 GHz. To verify the performance of the algorithm, we selected the publicly available star identification dataset (https://github.com/LiJianS/Star-identification-dataset-in-autonomous-celestial-navigation), which is used in 2019 China Post-Graduate Mathematic Contest in Modeling. The dataset consists of two parts. The first part is the predefined on-board star catalog whose star magnitude threshold is 6 Mv. There are 4908 stars in this star catalog, each of which contains four properties: “guide star number”, “right ascension”, “declination” and “magnitude”. The second part of the dataset provides four real observed star maps after the centroid estimation. The attribute of the observed star map is the coordinate of the centroid of stars: “*X*-coordinates” and “*Y*-coordinates”. Figure 7 shows the data attributes of the MVDT-SI algorithm used in the guide star catalog and the observed star map during the star identification.

### 5.2. Experiments on the Simulated Star Map

Based on the Section 4.1, we can calculate the projections of all stars on the star sensor in the guide star catalog. At this time, we can get the coordinates of each star of the projected star map. Based on the above projected star maps, we randomly selected 100 maps and added noises to verify the performance of our algorithm. To consider the influence of the number of stars in the simulated map on our algorithm, we verified it in the FOV of $12^{\circ }\times 12^{\circ }$ and $16^{\circ }\times 16^{\circ }$ respectively.

Also, in order to verify the robustness of our algorithm and prove that our algorithm is not affected by the focal length of star sensor and the shooting angle of star map, we generated 100 simulated maps based on the above projected star maps through the following steps.

(1) We randomly expanded or reduced the star coordinates on the simulated star map by 1–5 times.

(2) We randomly discarded φ star points on the simulated star map to simulate the interference of “missing star”, and randomly generated σ star points on the simulated star map to simulate the interference of “false star”. After completing the above steps, we marked the “false star” points in the simulated map as “−1”.

(3) We randomly rotated the simulated star map 0∼$360^{\circ }$ to simulate the different shooting angles of the star sensor.

**Figure 8:**
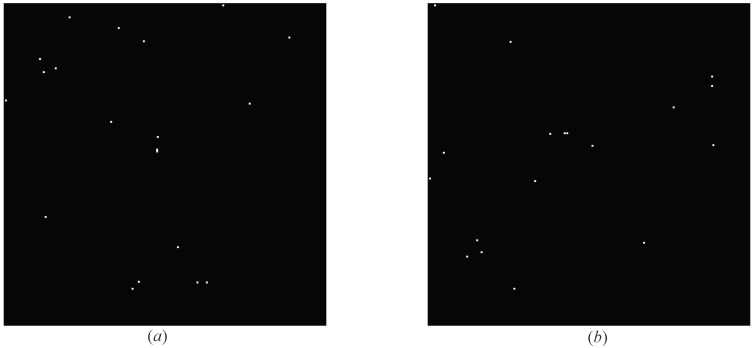
The difference between the original star map and the simulated star map. (**a**) The original star map. (**b**) The simulated star map.

It can be seen from the Figure 8 that there are differences in angles and number of stars between the original star map and the simulated star map.

#### 5.2.1. Results without False/Missing Stars

In an ideal case scenario, the observed star map doesn’t have the problem of false/missing stars (σ=0 and φ=0). Table 1 and Table 2 summarizes the performance of the star identification algorithms in the ideal case scenario with different values of FOV.

As can be seen from the results in Table 1 and Table 2, the identification accuracy of the MVDT-SI (λ=N) algorithm outperforms those of compared star identification algorithms. In addition, the SVST-SI algorithm is the fastest, but its identification accuracy is unacceptable. At the same time, we noticed that when we adjusted the hyperparameter λ of the MVDT-SI algorithm to 10, its accuracy is only reduced by 0.24% and 1.27% compared with λ=N, but its identification time is significantly reduced. When λ=10, the MVDT-SI algorithm can achieve more than 98% identification accuracy in only 0.11 s. If we need higher identification accuracy to meet real-world needs, the MVDT-SI algorithm can be achieved by further increasing the value of λ. Compared with the other three algorithms, the MVDT-SI (λ=N) algorithm is the fastest. Although in practical applications, storing an algorithm up to 2 MB is not a problem in the on-chip memory, the storage size is still an important factor to consider. The storage size of the feature of guide star catalog of each algorithm is shown in Table 1 and Table 2. Compared with the GMV, STOD and SISS algorithms, the MVDT-SI algorithm needs the least storage size.

#### 5.2.2. Results with False/Missing Stars Only

**Figure 9:**
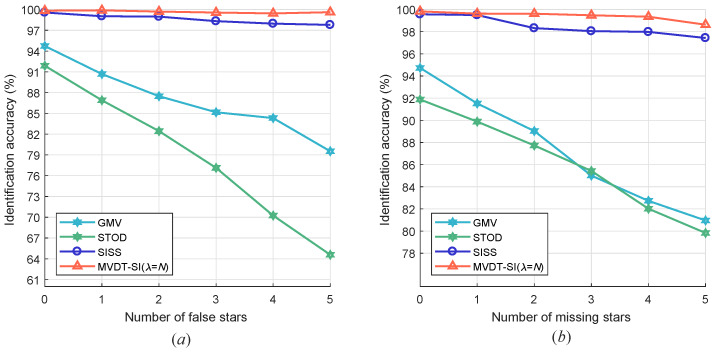
Performance of star identification algorithms in the scenario of (**a**) “false stars” or (**b**) “missing stars” when the FOV is $16^{\circ }\times 16^{\circ }$.

As was described in Section 5.1 of this paper, there are always “false stars” or “missing stars” in the observed star map. The performance of the star identification algorithms for the case of “false star” or “missing star” is shown in Figure 9. In the case of false stars or missing stars added to the observed star map, GMV and STOD will fail drastically. In this scenario, GMV, which is based on voting of the Euclidean distances of the neighboring stars from the reference star fails because the vote count for the reference star reduces due to the missed neighboring stars. STOD fails as well because it searches the tree database with the number of neighboring stars as the parameter. The SISS algorithm and our algorithm maintains a high robustness in this case. It can be seen that our MVDT-SI (λ=N) algorithm has stronger algorithm robustness.

#### 5.2.3. Results with Both False and Missing Stars

In some cases, there may be both false stars and missing stars in the observed map. As can be seen from Table 3, when there are multiple false stars and missing stars at the same time, the MVDT-SI (λ=N) algorithm still achieve more than 98.47% identification accuracy. When there are only missing stars or false stars (σ=0 or φ=0), the identification accuracy of the MVDT-SI (λ=N) algorithm can reach over 99.56% in any FOV ($12^{\circ }\times 12^{\circ }$ or $16^{\circ }\times 16^{\circ }$), which shows that the algorithm has good robustness.

### 5.3. Experiments on the Real Star Map

**Figure 10:**
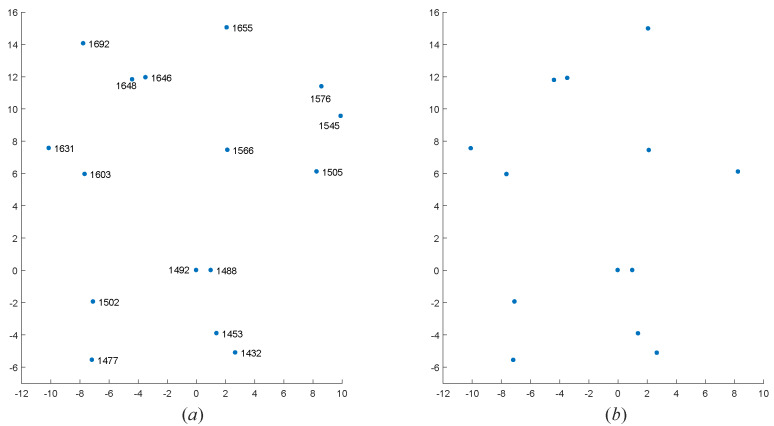
The identification results of our MVDT-SI algorithm on the first real star map. (**a**) The identification results (star numbers) of our MVDT-SI algorithm in the guide star catalog. (**b**) The real observed map of star sensor after standardization.

**Figure 11:**
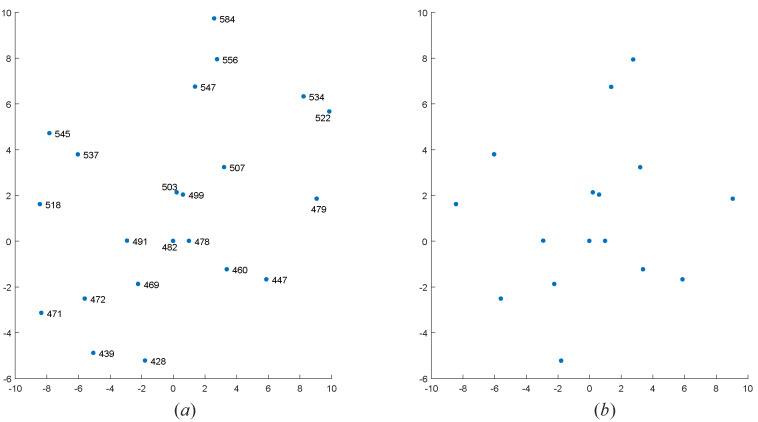
The identification results of our MVDT-SI algorithm on the second real star map. (**a**) The identification results (star numbers) of our MVDT-SI algorithm in the guide star catalog. (**b**) The real observed map of star sensor after standardization.

**Figure 12:**
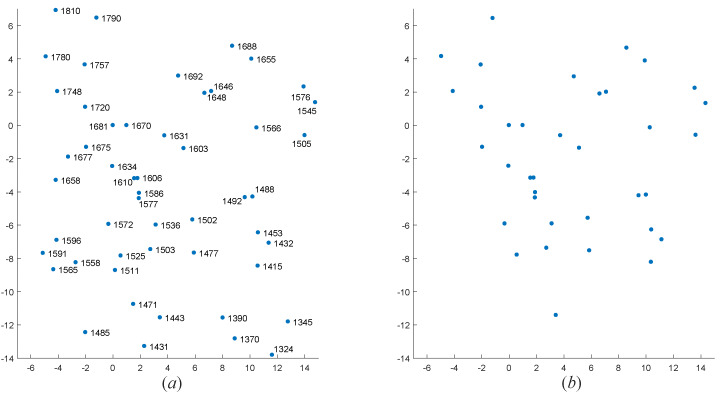
The identification results of our MVDT-SI algorithm on the third real star map. (**a**) The identification results (star numbers) of our MVDT-SI algorithm in the guide star catalog. (**b**) The real observed map of star sensor after standardization.

**Figure 13:**
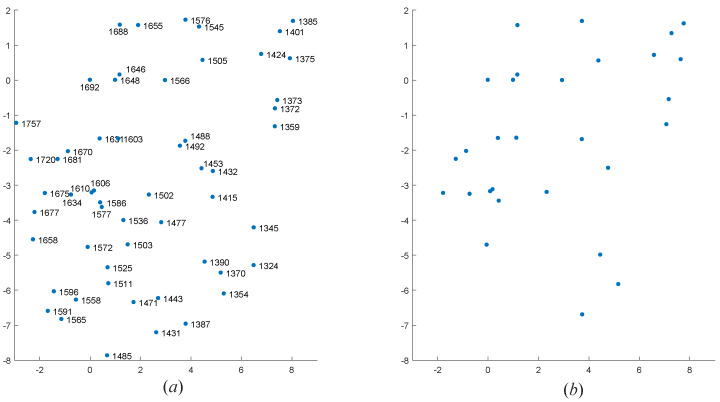
The identification results of our MVDT-SI algorithm on the fourth real star map. (**a**) The identification results (star numbers) of our MVDT-SI algorithm in the guide star catalog. (**b**) The real observed map of star sensor after standardization.

To further verify the effectiveness of the algorithm, we selected four real star maps for star identification base on the above dataset. These four star maps are sampled from the data of the real night sky. We only know the coordinates of centroid of star points in each observed star map. The FOV of star sensor recorded the first two star maps is $12^{\circ }\times 12^{\circ }$, and the number of pixels is 512×512; the FOV of star sensor recorded the last two star maps is $20^{\circ }\times 20^{\circ }$, and the number of pixels is 1024×1024. To visualize the results of the algorithm, we rotated and standardized those coordinates of centroid of star points. The star identification results are shown in Figure 10, Figure 11, Figure 12 and Figure 13.

As we can see, even if there are many noise points in the four observed star maps, our MVDT-SI algorithm can still successfully complete the identification.

In summary, the experimental results show that our MVDT-SI algorithm successfully identifies the observed star map without the magnitude information in both synthetic and real star maps and has good robustness. Compared with other star identification algorithms, the MVDT-SI algorithm has higher identification accuracy, shorter identification time and smaller storage size.

## 6. Conclusions

In this paper, a multi-view double-triangle algorithm for star identification is proposed. When a star sensor operates in “Lost-In-Space (LIS)” mode and the magnitude information of the observed map is lost, the MVDT-SI algorithm can still overcome the problem of “false stars” and “missing stars”, and identify the observed star map in a short time. Moreover, it is worth noting that when the number of stars of the observed star map is very small (N < 10) and there are a large number of false/missing stars (>50%) in the observed star map at the same time, the performance of our MVDT-SI algorithm might decline. To overcome this weakness, the field of views of the star sensor should be increased.

The MVDT-SI algorithm contains four steps. First, the coordinate system of the guide star catalog is transformed to obtain the two-dimensional coordinate representation of each guide star under different reference stars. Then, in order to ensure the coordinates and features of the observed star map are not affected by the shooting angle of the star sensor, we defined the rules for determining the coordinate system of the observed star map. Then, we constructed the multi-view double-triangle features for the observed star map and guide star features for the guide star catalog. Finally, by comparing the differences between their features, we obtained the number of each star point on the observed star map.

Experimental results show that our MVDT-SI algorithm has better robustness and shorter identification time under the interference of “false star” noises and “missing star” noises. In addition, experimental results show that our MVDT-SI algorithm can be applied to real star maps, thus it could be embedded in real autonomous navigation systems in future.

## Figures and Tables

**Table 1:** Benchmarking of star identification algorithms in the ideal case when the FOV is $12^{\circ }\times 12^{\circ }$.

Technique	Identification Accuracy (%)	Identification Time (s)	Storage Size (MB)
GMV [28]	92.97	0.758	1.31
STOD [27]	91.26	0.415	1.40
SISS [29]	99.08	0.420	1.70
SVST-SI	27.72	0.031	0.23
**MVDT-SI (λ=10)**	**99.51**	**0.102**	**0.44**
**MVDT-SI (λ=N)**	**99.75**	**0.285**	**0.44**

**Table 2:** Benchmarking of star identification algorithms in the ideal case when the FOV is $16^{\circ }\times 16^{\circ }$.

Technique	Identification Accuracy (%)	Identification Time (s)	Storage Size (MB)
GMV [28]	94.73	0.767	1.31
STOD [27]	91.88	0.412	1.40
SISS [29]	99.57	0.501	1.70
SVST-SI	17.36	0.034	0.23
**MVDT-SI (λ=10)**	**98.56**	**0.112**	**0.44**
**MVDT-SI (λ=N)**	**99.83**	**0.483**	**0.44**

**Table 3:** Identification accuracy of MVDT-SI(λ=N) algorithm in the scenario of “false stars” and “missing stars” with different values of the FOV ($12^{\circ }\times 12^{\circ }$ and $16^{\circ }\times 16^{\circ }$).

φ	0	1	2	0	1	2
σ	$12^{\circ }\times 12^{\circ }$			$16^{\circ }\times 16^{\circ }$		
0	99.75%	99.67%	99.64%	99.83%	99.63%	99.62%
1	99.88%	99.72%	99.52%	99.88%	99.69%	99.67%
2	99.56%	98.54%	98.47%	99.70%	99.43%	99.44%
